# Spanish experts consensus on emergency psychiatric care in hospital emergency departments

**DOI:** 10.1186/s12888-024-05939-1

**Published:** 2024-07-04

**Authors:** Rafael Manuel Gordillo-Urbano, Benedicto Crespo-Facorro, Víctor Pérez-Solá, Narcís Cardoner, Elena García-Ligero, Carmen Moreno, Josep Antoni Ramos-Quiroga, Miguel Ruiz-Veguilla, Mireia Vázquez-Vallejo, Juan Luis Prados-Ojeda, the Spanish Expert Group in Emergency Psychiatric Care

**Affiliations:** 1 Mental Health Clinical Management Unit of the South Health Management Area of Cordoba (Unidad de Gestión Clínica de Salud Mental del Área de Gestión Sanitaria Sur de Córdoba). Hospital Infanta Margarita, Córdoba, Spain; 2 Department of Mental Health, Hospital Universitario Virgen del Rocío, Seville, Spain; 3 Department of Psychiatry, School of Medicine, University of Sevilla, Seville, Spain; 4 Institute of Biomedicine of Seville (Instituto de Biomedicina de Sevilla; IBiS), Seville, Spain; 5 Spanish Network for Research in Mental Health (Centro de Investigación Biomédica en Red Salud Mental; CIBERSAM), Health Institute Carlos III (Instituto de Salud Carlos III; ISCIII), Madrid, Spain; 6 Mental Health Institute. Hospital del Mar, Barcelona, Spain; 7 Hospital del Mar Medical Research Institute, Barcelona, Spain; 8 University Pompeu Fabra, Barcelona, Spain; 9 Sant Pau Biomedical Research Institute, Hospital de La Santa Creu I Sant Pau, Psychiatry and Legal Medicine, UAB, CIBERSAM, Barcelona, Spain; 10 Department of Psychiatry and Forensic Medicine, School of Medicine Bellaterra, Universitat Autònoma de Barcelona, Barcelona, Spain; 11 Department of Child and Adolescent Psychiatry, Institute of Psychiatry and Mental Health, Hospital General Universitario Gregorio Marañón, IiSGM, CIBERSAM, ISCIII, School of Medicine, Universidad Complutense, Madrid, Spain; 12 Department of Mental Health, Hospital Universitari Vall d’Hebron, Barcelona, Spain; 13 Group of Psychiatry, Mental Health and Addictions, Vall d’Hebron Research Institute (Vall d’Hebron Institut de Recerca; VHIR), Barcelona, Spain; 14 Department of Psychiatry and Forensic Medicine, Universitat Autònoma de Barcelona, Barcelona, Spain; 15 Psychiatry and Psychology Service, Hospital Clinic, Barcelona, Spain; 16 Department of Mental Health, Hospital Universitario Reina Sofía, Córdoba, Spain; 17 Morphological and Socio-Sanitary Sciences Department, School of Medicine, University of Córdoba, Córdoba, Spain; 18 Maimonides Biomedical Research Institute of Cordoba (Instituto Maimónides de Investigación Biomédica de Córdoba; IMIBIC), Córdoba, Spain

**Keywords:** Emergency service, Hospital, Emergency services, Psychiatric, Quality Improvement, Consensus, Delphi technique

## Abstract

**Background:**

The demand for urgent psychiatric care is increasing, but in Spain there are no clear recommendations for emergency departments (ED) on how to optimize care for patients with psychiatric emergencies. We aimed to provide expert consensus recommendations on the requirements for general hospitals´ emergency departments to treat patients with urgent psychiatric symptoms.

**Methods:**

We used a modified Delphi technique. A scientific committee compiled 36 statements based on literature search and clinical experience. The statements covered the organizational model, facilities, staffing, safety, patient interventions, and staff training. A panel of 38 psychiatry specialists with expertise in psychiatric emergencies evaluated the questionnaire in two rounds.

**Results:**

After two rounds of voting, 30 out of 36 proposed items (83%) were agreed upon. The panel agreed that psychiatric emergencies should be managed in a general hospital, with dedicated facilities for patient assessment, direct supervision of patients at risk, and an observation unit run by the psychiatric service. In addition to the psychiatrist, the ED should have specialist nurses and security staff available 24/7. Social workers should also be readily available. ED and consulting rooms should be designed to ensure patient and staff safety. A triage system should be established for patients with psychiatric symptoms, with medical evaluation preceding psychiatric evaluation. Guidance on supplies, equipment, and staff training is also provided.

**Conclusion:**

All ED in general hospitals should have adequate resources to handle any psychiatric emergency. This paper provides recommendations on the minimum requirements to achieve this goal.

**Supplementary Information:**

The online version contains supplementary material available at 10.1186/s12888-024-05939-1.

## Introduction

The Spanish National Health Service (NHS) hospitals attend over 23.5 million emergencies annually, with hospital emergency departments (ED) being visited 0.50 times per person per year [1]. Emergency attendance at specialised care level has shown a clear upward trend in almost all Spanish territories in the last few years [1]. There is a high and growing demand for emergency care related to mental health or substance use problems [2–4]. This increase is remarkable in both child and adolescent population [5]. Psychiatric problems may account for about 5% of all ED visits [6].

Access to high-quality psychiatric emergency care is an essential component of a comprehensive medical system [7]. Hospitals and community psychiatric facilities should provide emergency psychiatric care comparable to the care provided for other medical emergencies [7]. Unfortunately, there are often more emergency psychiatry needs than available resources, which can result in mishandling psychiatric emergencies and deterioration of patients' conditions [2]. A variety of emergency mental healthcare models have been developed in different countries to address the growing need for high-quality psychiatric emergency care; however, evidence to guide best practices and organizational structure of comprehensive psychiatric emergency services is scarce [2]. Guidelines and recommendations exist for the organisation of psychiatric emergency care [7–18], but models are very different and cannot be directly applied to every setting. In Spain, there are no clear recommendations on the characteristics and requirements that hospital emergency department should have to provide optimal care for patients with psychiatric emergencies.

In this study we aimed to provide recommendations based on an expert consensus on the minimum requirements that the hospitals attending psychiatric emergencies should have in their emergency department. This includes issues related to the organizational model, facilities, staff, safety, patient interventions, and staff training. The project arose from the initiative of the Spanish Society of Psychiatric Emergencies (Sociedad Española de Urgencias Psiquiátricas; SEDUP [19]) and has been endorsed and declared to be of scientific interest by the Spanish Society of Psychiatry and Mental Health (Sociedad Española de Psiquiatría y Salud Mental; SEPSM) [20].

## Methods

### Design

In this project, a modified Delphi method was used following RAND/UCLA recommendations [21, 22]. A scientific committee of 10 experts in psychiatric emergencies led the study. After a search and review of available literature from various sources, and considering their expertise, the scientific committee generated 36 debatable statements addressing the minimum requirements that hospitals attending psychiatric emergencies should have to provide adequate care to patients with mental health-related problems. In a second step, these statements were sent to a panel of psychiatrists with expertise in psychiatric emergencies for an online evaluation and validation by voting in two rounds.

### Literature search

A PubMed literature search was performed with focus on guidelines, consensus and reviews addressing models of hospital care for psychiatric emergencies. The search strategy included the following terms: Emergency Psychiatric Services; Psychiatric Emergencies; Patient care; Quality of Health Care; Quality Assurance, Health Care; Standard of Care; Triage; Critical Pathways; Books and Documents; Guidelines; Consensus; Reviews. The literature search was conducted in October 2022. It was restricted to articles published in English and Spanish during the preceding ten years. In addition, the websites of leading Spanish, European, and American psychiatric or emergency medicine scientific societies were searched: SEDUP [19]; SEPSM [20]; European Psychiatrists Association (EPA) [23]; Royal College of Psychiatrists (RCPysch) [24]; Royal College of Emergency Medicine (RCEM) [25]; American Psychiatric Association (APA) [26]; American Association for Emergency Psychiatry (AAEP) [27]; American College of Emergency Physicians (ACEP) [28]; and the American Academy of Emergency Medicine (AAEM) [29].

### Statements development

The scientific committee qualitatively reviewed the literature and in total 36 statements were agreed upon by all the experts. The statements were divided into the following six blocks: 1) Model of care; 2) Facilities; 3) Staff; 4) Staff and patient safety; 5) Patient interventions; and 6) Training.

### Panellists

Panellists were selected by the scientific committee applying a snowball sampling technique and considering the following selection criteria: they should 1) be psychiatry specialists; 2) be members of the SEDUP or the SEPSM; 3) have experience in managing psychiatric emergencies. An attempt was made to involve panellists from all the Autonomous Communities of Spain.

### Delphi rounds and analysis

Panellists completed the questionnaire in two rounds using a web-based survey that was created in-house. Participants were allowed to provide feedback if they found the statements unclear, which would be considered during the article discussion. Panellists used a 9-point Likert scale (1: complete disagreement; 9: entire agreement) to assess each statement. Responses were organised into three groups: 1–3 were considered as disagreement, 4–6 as neither agreement nor disagreement and 7–9 as agreement. Consensus was reached if 1) the median of the responses was in the 7–9 range (agreement) or 1–3 (disagreement), 2) less than one-third of the panellists voted outside these ranges, and 3) the interquartile range (IQR) was less than 4  (Tables 1, 2, 3, 4 and 5).

**Table 1:** Results of the items about the model of care and facilities

	**Median (IQR)**	**Degree of agreement**	**Results**
1. In general, in Spain, hospital emergency departments are adequately designed for the care of psychiatric emergencies	5 (4–5)	79%	No consensus
2. Psychiatric emergencies should be attended to within the space of a general hospital	9 (9–9)	95%	Agreement in 1st round
3. The care of psychiatric emergencies within a psychiatric speciality hospital may have limitations compared to care in a general hospital	9 (8–9)	97%	Agreement in 1st round
4. Psychiatric services should have a specific psychiatric emergency unit with a physician responsible for psychiatric care in the emergency department	9 (7–9)	77%	Agreement in 1st round
In the emergency departments of general hospitals, it should be required:			
5. A separate waiting room for patients with psychiatric symptoms	7 (5–8)	53%	No consensus
6. A specific room for the assessment of patients with psychiatric symptoms	9 (8–9)	95%	Agreement in 1st round
7. A room with the possibility of direct supervision for patients at risk of suicide, agitation, aggression or in need of mechanical restraint	9 (9–9)	100%	Agreement in 1st round
8. An observation/short-stay unit	9 (7–9)	92%	Agreement in 1st round
9. The observation or short-stay unit should be run by the psychiatric service	8 (5–9)	68%	Agreement in 2nd round
10. The observation or short-stay unit should be in charge of the general emergency department with referral to psychiatry	5 (2–8)	29%	No consensus
11. Facilities for the care of psychiatric emergencies in children and adolescents (especially if they have intellectual disabilities or neurodevelopmental disorders) should have spaces with low sensory stimulation and the accompaniment of family members should be always allowed	9 (9–9)	100%	Agreement in 1st round

*IQR* Interquartile range

**Table 2:** Results of the items about staffing

	**Median (IQR)**	**Degree of agreement**	**Results**
In addition to the psychiatrist, in the emergency departments of general hospitals, the 24-h presence of the following staff should be required:			
1. A specialist child and adolescent psychiatrist for the urgent care of these patients	8 (5–9)	58%	No consensus
2. Nursing staff or advanced practice nurses attached to the Psychiatric Service on a 24-h basis	9 (8–9)	85%	Agreement in 1st round
3. A clinical psychologist 24 h a day	3 (1–5)	58%	No consensus
4. In the emergency departments of general hospitals, access to social work in the morning and afternoon shifts or in less than 24 h should be guaranteed	9 (7–9)	84%	Agreement in 2nd round
5. In the emergency departments of general hospitals, 24-h presence of security staff in sufficient numbers and with specific training in psychiatric emergencies should be required	9 (8–9)	95%	Agreement in 2nd round

*IQR* Interquartile range

**Table 3:** Results of the items about staff and patient safety

	**Median (IQR)**	**Degree of agreement**	**Results**
1. Security measures at the entrance to the emergency department for the detection of weapons or potentially dangerous objects are recommended	8 (6–9)	69%	Agreement in 1st round
2. The installation of closed-circuit television in the entire emergency department area is necessary	8 (6–9)	74%	Agreement in 2nd round
3. There should be a panic button or alarm system in the consulting rooms	9 (9–9)	100%	Agreement in 1st round
4. Consulting rooms should be appropriately designed and furnished to preserve patient and staff safety	9 (9–9)	100%	Agreement in 1st round
5. There should be an observation window that allows the patient's condition to be checked from the outside, but still provides a sufficient degree of privacy	9 (8–9)	92%	Agreement in 1st round

*IQR* interquartile range

**Table 4:** Results of the items about interventions on the patient

	**Median (IQR)**	**Degree of agreement**	**Results**
1. There should be a specific triage system for patients with psychiatric symptoms	9 (5–9)	74%	Agreement in 2nd round
2. Protocols should be in place that clearly define what constitutes a psychiatric urgency and emergency	9 (9–9)	97%	Agreement in 1st round
3. There should be clear protocols for situations where professionals from different medical specialties need to be involved	9 (9–9)	97%	Agreement in 1st round
4. All patients with psychiatric symptoms should always receive an initial evaluation by an emergency physician to identify and stabilise any medical conditions that may contribute to the psychiatric symptoms	9 (6–9)	74%	Agreement in 1st round
5. Patients with chronic psychiatric pathologies presenting with a similar episode to previous ones should receive an initial assessment by an emergency physician	8 (5–9)	74%	Agreement in 2nd round
6. Psychiatric care should always be provided in the absence of psychoactive substance intoxication	9 (7–9)	79%	Agreement in 1st round
7. A maximum time limit should be defined for certain psychiatric emergencies (agitation, psychosis, suicidal ideation, or gesture, etc.)	7 (5–9)	58%	No consensus
8. Family accompaniment of patients with psychiatric symptoms during their stay in the Emergency Department should be permitted and compulsory in the case of minors	9 (7–9)	85%	Agreement in 1st round
9. Access to complementary laboratory tests on blood, urine, and CSF (including screening for toxicants and plasma drug levels) should be available	9 (9–9)	100%	Agreement in 1st round
10. Access to complementary neuroimaging tests should be available	9 (9–9)	97%	Agreement in 1st round
11. Access to electroencephalogram must be available	9 (6–9)	72%	Agreement in 1st round
12. A basic psychopharmaceutical kit should be available for use in emergency care	9 (9–9)	100%	Agreement in 1st round

*CSF* Cerebrospinal fluid; IQR: interquartile range

**Table 5:** Results of the items about training

	**Median (IQR)**	**Degree of agreement**	**Results**
1. Regular training in psychiatric emergency medicine (including its legal framework, cultural differences, diversity, and stigma-related issues) is needed for all healthcare staff working in the emergency department, especially for those new to the service	9 (9–9)	100%	Agreement in 1st round
2. There is a need for joint clinical sessions between psychiatrists attending psychiatric emergencies and emergency department physicians	9 (9–9)	100%	Agreement in 1st round
3. Resident physicians in the team must practise under the supervision of a staff member of the Psychiatry Service	9 (9–9)	95%	Agreement in 1st round

I*QR* Interquartile range

The results of the first round were analysed, and any statements without consensus were voted again in a second round. Two statements (items 4 and 5, Table 2) were rephrased between the two rounds following suggestions from the panellists to make them more specific. Before the second round, the panellists reviewed the personal and global questionnaire results, along with anonymous individual comments. This allowed them to compare their opinions with their peers and potentially adjust their initial responses. The second-round results were analysed using the same criteria as the first round ones.

Results are shown in tables as median and IQR of the panellists’ responses and degree of agreement, which was defined as the percentage of panellists who voted within the category containing the median answer (1–3, 4–6 or 7–9) (Tables 1, 2, 3, 4 and 5). Considering the consensus items, the scientific committee developed a table summarising the recommendations (Table 6).

**Table 6:** Summary of recommendations

Model of care	Psychiatric emergencies should be attended to within the space of a general hospital Psychiatric services should have a specific psychiatric emergency unit with a physician responsible for psychiatric care in the emergency department
Facilities	In the emergency departments of general hospitals, the following facilities should be required: A specific room for the assessment of patients with psychiatric symptoms A room with the possibility of direct supervision for patients at risk of suicide, agitation, aggression or in need of mechanical restraint An observation/short-stay unit run by the psychiatric service Facilities for the care of psychiatric emergencies in children and adolescents (especially if they have intellectual disabilities or neurodevelopmental disorders) should have spaces with low sensory stimulation and the accompaniment of family members should be always allowed
Staff	In the emergency departments of general hospitals, in addition to the psychiatrist, the presence of the following staff should be required: Nursing staff or advanced practice nurses attached to the Psychiatric Service on a 24-h basis Social work staff in the morning and afternoon shifts or in less than 24 h Security staff in sufficient numbers and with specific training in psychiatric emergencies on a 24-h basis
Staff and patient safety	Security measures at the entrance to the emergency department for the detection of weapons or potentially dangerous objects are recommended The installation of closed-circuit television in the entire emergency department area is necessary There should be a panic button or alarm system in the consulting rooms Consulting rooms should be appropriately designed and furnished to preserve patient and staff safety There should be an observation window that allows the patient's condition to be checked from the outside, but still provides a sufficient degree of privacy
Interventions on the patient	Triage system: There should be a specific triage system for patients with psychiatric symptoms Protocols: Protocols should be in place that clearly define what constitutes a psychiatric urgency and emergency There should be clear protocols for situations where professionals from different medical specialties need to be involved Initial assessment: All patients with psychiatric symptoms should always receive an initial evaluation by an emergency physician to identify and stabilise any medical conditions that may contribute to the psychiatric symptoms Patients with chronic psychiatric pathologies presenting with a similar episode to previous ones should receive an initial assessment by an emergency physician Psychiatric care should always be provided in the absence of psychoactive substance intoxication Family accompaniment: Family accompaniment of patients with psychiatric symptoms during their stay in the Emergency Department should be permitted and compulsory in the case of minors Resources: Access to laboratory tests on blood, urine, and cerebrospinal fluid (including screening for toxicants and plasma drug levels), neuroimaging, electroencephalogram, and a basic psychopharmaceutical kit must be available
Training	Regular training in psychiatric emergency medicine is needed for all healthcare staff working in the emergency department, especially for those new to the service Regular training should include psychiatric emergency medicine legal framework, cultural differences, diversity, and stigma-related issues There is a need for joint clinical sessions between psychiatrists attending psychiatric emergencies and emergency department physicians Resident physicians in the team must practise under the supervision of a member of the psychiatry department staff

## Results

Thirty-nine panellists were invited to participate in the study; 39 responded to the first round, and 38 responded to both rounds of the Delphi. Of these, 92% were under 50, 53% were women, 79% had more than five years of professional experience as psychiatrists, and 74% had more than five years of clinical practice attending psychiatric emergencies (not counting residency). There were panellists from 13 Autonomous Communities and the city of Ceuta. Ninety-five per cent were currently treating psychiatric emergencies, 64% more than 20 per month, 100% attended psychiatric emergencies in general hospitals, and 97% in the public health system.

In the first round, 24 items were agreed upon. In the second round, six additional items reached consensus. Out of the 36 proposed items, 30 (83%) were agreed upon after two rounds of voting. All items that reached consensus were consensual in agreement and none in disagreement. The scores for all items and the consensus results are detailed in Tables 1, 2, 3, 4 and 5 and summarised in Table 6.

### Model of care, facilities, and staffing

The recommendations agreed upon in this section are shown in Tables 1 and 2.

It was considered that psychiatric emergencies should be managed within a general hospital and that psychiatric services should have a specific psychiatric emergency unit with physicians responsible for psychiatric care in the ED. There was no consensus on whether hospital EDs in Spain are adequately designed for psychiatric emergency care.

The panel agreed that the facilities required in the EDs of general hospitals are: 1) a specific room for the assessment of patients with psychiatric symptoms; 2) a room with the possibility of direct supervision for patients at risk of suicide, agitation, aggression or in need of mechanical restraint; and 3) an observation/short-stay unit run by the psychiatric service. There was no agreement on whether a separate waiting room for patients with psychiatric symptoms was required. Additionally, the panel agreed that facilities for the care of psychiatric emergencies in children and adolescents should have spaces with low sensory stimulation and that family members should always be allowed to accompany the patients.

In the EDs of general hospitals, in addition to the psychiatrist, the panel considered that the following staff should be present: 1) nursing staff or advanced practice nurses attached to the Psychiatric Service on a 24-h basis; 2) social work staff in the morning and afternoon shifts or in less than 24 h; and 3) security staff in sufficient numbers and with specific training in psychiatric emergencies on a 24-h basis. There was no consensus on whether a specialist child and adolescent psychiatrist or a 24-h clinical psychologist would be necessary.

### Staff and patient safety

The recommendations agreed upon in this section are summarised in Table 3 and include the need for security measures at the entrance of the ED for the detection of weapons or potentially dangerous objects, the installation of a closed-circuit television system in the whole area of the ED and a panic button or alarm system in the consulting rooms, which should be appropriately designed and furnished to ensure patient and staff safety.

### Patient interventions

The recommendations agreed upon in this section are summarised in Table 4. They include guidance on the triage system, which should be specific for patients with psychiatric symptoms, protocols to be implemented, the initial assessment of patients with psychiatric symptoms, which should always be performed by an emergency physician, family accompaniment, and access to resources, which should include tests on blood, urine, and cerebrospinal fluid (including screening for toxicants and plasma drug levels), neuroimaging tests, electroencephalogram and a basic psychopharmaceutical kit. There was no agreement on setting a maximum time limit (meaning time from a patient's arrival at the ED until they receive care) for specific psychiatric emergencies such as agitation, psychosis, suicidal ideation, or gestures.

### Training

The recommendations agreed upon in this section are summarised in Table 5.

The panel agreed that all healthcare staff working in the ED should have regular training in psychiatric emergency medicine, including legal frameworks, cultural differences, diversity, and stigma-related issues. Especially new employees in the ED require this training. Joint clinical sessions between psychiatrists attending psychiatric emergencies and ED physicians are necessary. Additionally, the team's resident doctors should practice under the supervision of a member of the psychiatry department staff.

## Discussion

In this consensus, a panel of psychiatrists specialising in psychiatric emergencies developed recommendations based on a literature review and their clinical expertise, and outlined the minimal requirements that EDs in general hospital should have to ensure proper care for patients with psychiatric symptoms.

The NHS in Spain receives over 50 million emergency care requests annually, which are addressed by three resources: primary care, mobile emergency services, and hospital EDs. The latter account for almost half of the total urgent consultations (23.5 million) [1, 30]. In 2019, over 50% of the Spanish population sought emergency medical services, primarily for minor conditions [1, 30]. Considering that up to 5% of emergency consultations are mental health-related, maintaining efficient and quality hospital emergency services can be challenging [2, 6].

In Spain, the healthcare system is decentralised with national coordination. The NHS provides universal coverage and is primarily funded through taxation. Although the Ministry of Health is responsible for national planning and regulation, the 17 regional health authorities have primary jurisdiction over operational planning, resource allocation, purchasing, and provision of healthcare services [31]. The National Institute of Health Management (Instituto Nacional de Gestión Sanitaria; INGESA) is responsible for health services in the cities of Ceuta and Melilla [32]. This decentralised model results in varying models of health care among regions. Implementing consensus recommendations like the ones we present, could improve emergency care for patients with mental disorders and promote equality in the system. In addition, in Spain, individuals can freely choose their preferred medical care without any cost based on their subjective perception of symptoms severity. A specialist does not screen most patients who come to the ED with psychiatric symptoms beforehand, so it is essential to implement a model that permits the handling of a large volume of patients and offers fast and quality care. In our study, there was a wide range of responses to whether hospital EDs are well designed for psychiatric emergency care, suggesting that the service may not be optimal everywhere.

Regarding the model of care and facilities, hospital-based psychiatric emergency services are usually structured in one of two ways: either as a consultation service for the general ED or as a specialised mental health unit, located within the ED or in a separate facility nearby [2]. In our consensus, the panellists were in favour of the second model. It was considered that psychiatric emergencies should be attended within a general hospital and that psychiatric services should have a specific psychiatric emergency unit with a physician responsible for psychiatric care in the ED. In this regard, it was proposed that there should be a specific room for the assessment of patients with psychiatric symptoms, a room with the possibility of direct supervision for patients at risk of suicide, agitation, and aggression or in need of mechanical restraint and an observation/short-stay unit run by the psychiatric service. This recommendation aligns with guidelines proposing that an appropriate area should be available in the ED to observe patients with mental health issues. The site should be safe, calm and quiet [17]. The need for a specific waiting room for patients with psychiatric pathologies sparked a debate among the panellists. Some argued that it could increase stigma, while others saw potential benefits. No consensus was reached on this issue. Additionally, the panel agreed that the psychiatric emergencies care within a psychiatric speciality hospital might have limitations compared to the care in a general hospital.

Children and adolescents are increasingly seeking mental health services, comprising a population of special concern [11]. The panel considered that psychiatric emergency facilities for children and adolescents should have low sensory stimulation spaces, especially for those with intellectual disabilities or neurodevelopmental disorders. Family members should always be allowed to accompany them.

Regarding staffing, there was no agreement on whether to always have a specialist for child and adolescent psychiatric disorders and a clinical psychologist present in the ED. In the comments, some panellists considered that, although they are professionals who could be valuable in some cases, their presence 24 h a day is optional. The speciality of child and adolescent psychiatry has only recently been created in Spain [33]. Therefore, it is not possible to have specialists in all hospital emergency departments. In any case, we consider that the psychiatry specialist should be qualified to attend emergencies in this population. However, a subsequent assessment by a specialist in child and adolescent psychiatry is advisable. Similarly, psychiatrists can fulfil the role of psychologists in emergencies with the advantage that they can prescribe the necessary medication for a psychiatric crisis. Therefore, as agreed by the panellists, the necessary 24-h professionals would include psychiatrists, psychiatric nurses, security staff trained in psychiatric emergencies, and social workers available during the morning and afternoon shifts or within 24 h. This recommendation aligns with urgent psychiatric hospital care models in other countries [2]. In Spain, mental health nursing is a specialized field, but the availability of such professionals is limited. Ideally, all emergency departments in general hospitals should have mental health nurses in the future. However, for now, it would be beneficial to have nurses working under the psychiatry service or advanced practice nurses.

The guidelines on safety in ED caring for patients with mental disorders include recommendations on providing access to assessment rooms suitable for conducting high-risk assessments [18]. The panellists provided generic safety recommendations, considering that consulting rooms should be adequately designed and furnished to ensure patient and staff safety. They emphasised the need for a closed-circuit television installation in the ED, a panic button, and an observation window in consulting spaces. Closed-circuit television should be installed throughout the entire emergency department, not just in the psychiatric wards, to prevent stigmatization.

The panellists extensively commented on patients’ interventions, specifically on the triage system, which was agreed upon in the second round, and the acceptable waiting times. Regarding the triage, nurses commonly performed it in hospital EDs [17]. The panellists advocated for a specific triage system for patients with psychiatric symptoms. In this way, patients can receive proper mental health triage upon arrival to assess their risk of self-harm, suicide, or leaving the ED before treatment is complete and to determine the necessary level of observation during their ED stay [17]. However, patients with psychiatric symptoms still should undergo the regular triage process performed on all patients upon entering the ED. Regarding the waiting time until an assessment was performed, no consensus was reached. The panellists agreed with other authors that patients with psychiatric symptoms should undergo triage upon arrival based on an initial risk assessment and safety evaluation for both the patient and others. Patients with psychiatric symptoms requiring high-priority triage include those with active suicidal ideation, acute psychosis, violent, combative or homicidal behaviour, acute mania or acute agitation [13]. A health care provider should immediately evaluate these patients, and they should not be left alone or allowed to leave the ED before assessment [13]. Patients with psychiatric symptoms who are not at risk of harming themselves or others may receive standard triage [13]. This formal assessment may include patients with depression but without suicidal thoughts, stable patients with psychiatric symptoms seeking medication refills, or outpatient referrals [13].

The panellists agreed that an acute psychiatric emergency requires a medical evaluation (meaning an evaluation by an emergency clinician) before a non-urgent one, as recommended by other authors as well [13]. Rapid identification of health needs is critical when a patient presents to ED. For patients with mental illness, this is no exception [12]. The ED evaluation serves two purposes: to determine if a non-psychiatric illness is causing or making the psychiatric condition worse and to identify any acute non-psychiatric conditions that require immediate treatment and co-occur with the psychiatric issue [13]. However, some authors argue that if a patient has a long history of a psychiatric illness and presents a similar clinical picture with each episode, a multi-system health assessment may not be necessary [12]. The panel disagreed with the exception and agreed in the second round that also patients with chronic psychiatric pathologies who present with similar symptoms to previous episodes should receive an initial assessment by an emergency physician.

Finally, among other recommendations, the panellists agreed on the resources that should be accessible in the EDs of general hospitals and the training required for staff. These recommendations are also included in guidelines by other authors [9, 10, 14, 15, 18, 34]. It is crucial to emphasize the significance of collaboration between emergency physicians and psychiatrists when treating patients with psychiatric emergencies. To ensure effective treatment, it is essential to establish joint protocols and clinical sessions. Additionally, ED staff training should focus on psychiatric pathology, medico-legal issues, cultural sensitivity, and ethical aspects of patient care.

Our work has the inherent limitations of the Delphi method meaning that it is impossible to discuss the recommendations in depth or that there might have been some bias in the selection of the panellists. The limited number of child and adolescent psychiatrists who participated as panellist might have biased the recommendations made on the care of this population. However, the scientific committee had considered the panellists' comments when drafting the discussion and the choice of participants was prudent and included only physicians with proven experience in the field. Additionally, the panel of experts in this study included only psychiatrists. We believe that these specialists have the most accurate understanding of the care needs of individuals with psychiatric pathologies. However, it would also have been valuable to include other professionals such as other clinicians, nurses or social workers. We hope that this work can be the starting point for a broader interdisciplinary consensus in the future.

## Conclusions

In conclusion, all EDs in general hospitals should have the necessary resources to handle any psychiatric emergency. This includes harm-reducing facilities, adequate supplies and equipment, trained staff, and coordination with emergency physicians and social services. This paper outlines the minimum requirements for EDs to achieve this goal.

### Supplementary Information

Supplementary Material 1.

## Data Availability

The data that support the findings of this study are available from the corresponding author upon reasonable request.
