# Supplementary material for: Spanish experts consensus on emergency psychiatric care in hospital emergency departments
Source: BMC Psychiatry. 2024 Jul 4;24:489. doi: 10.1186/s12888-024-05939-1 (PMC11225242; doi:10.1186/s12888-024-05939-1)
Supplement: Supplementary file 1 — Supplementary Material 1. [file 12888_2024_5939_MOESM1_ESM.docx]

**Appendix 1. Members of the Spanish Expert Group in Emergency Psychiatric Care**

Francisco Arnau Peiró (Consorcio Hospitalario Provincial. Castellón); Sergio Arques Egea (Hospital Universitario Arnau de Vilanova-Lliria. Lliria, Valencia); Maria Luisa Barrigón Estévez (Hospital General Universitario Gregorio Marañón. Madrid); María Teresa Campillo Sanz (Parc de Salut Mar. Barcelona); Pilar Casallo Calderón (USMC Otero, Ceuta); María Ángeles Chacón Gamero (Hospital Universitario Virgen del Rocío. Sevilla); Ilene Adriana Criado Gutiérrez (Hospital Universitario de Canarias. La Laguna, Santa Cruz de Tenerife); Juan Jose de Frutos Guijarro (Hospital Can Misses. Ibiza); Elena Fernández León (Comunidad Terapéutica / ETIC Hospital Universitario Virgen del Rocío. Sevilla); Giovanna Fico (Hospital Clínic Barcelona. Barcelona); Fernando García Lázaro (Hospital Universitario Virgen del Rocío. Sevilla); Anna Giménez Palomo (Hospital Clínic Barcelona. Barcelona); Francisco Gotor Sánchez-Luengo (Hospital Universitario Virgen del Rocío. Sevilla); Isabel Hernández Otero (Hospital Clínico Universitario Virgen de la Victoria. Málaga); Carmen Iranzo Tatay (Hospital Universitario y Politécnico La Fe. Valencia); Marta López García (Hospital Álvaro Cunqueiro. Vigo, Pontevedra); Álvaro López Díaz (Hospital Universitario Virgen Macarena. Sevilla); Jorge López Álvaro (ESM Don Benito/Hospital de Mérida. Badajoz); Constanza Machio Paloma (Hospital General de Llerena. Llerena, Badajoz); Rebeca Méndez Iglesias (Hospital Marítimo de Oza. A Coruña); Cristina Merino del Villar (Hospital Can Misses, Ibiza); Patricio Molero Santos (Clínica Universidad de Navarra. Pamplona); Estanislao Mur Milá (Parc de Salut Mar – INAD. Barcelona); Nelson Naranjo Mendoza (Complejo Hospitalario Insular Materno Infantil. Las Palmas de Gran Canaria); Daniel Núñez Arias (Hospital Naval. Complejo Hospitalario Universitario de Ferrol. Ferrol, A Coruña); Clara Oliveras Salva (Hospital Clínic de Barcelona, Barcelona); Álvaro José Palma Conesa (Hospital La Merced, Osuna. Sevilla); José María Pelayo Terán (Hospital Universitario El Bierzo. Ponferrada, León); Amanda Rodríguez Urrutia (Hospital Universitari Vall d'Hebrón. Barcelona); Pedro Manuel Ruiz Lázaro (Hospital Clínico Universitario Lozano Blesa. Zaragoza); Salvador Ruiz Murugarren (Hospital Universitario Príncipe de Asturias. Alcalá de Henares. Madrid); María Sagué Vilavella (Hospital Clínic de Barcelona. Barcelona); Purificación Salgado Serrano (Hospital del Mar. Barcelona); Antonio Serrano García (Complejo Asistencial Universitario de León. León); Marina Sevilla Fernández (Hospital Universitario Virgen del Rocío. Sevilla); Javier Vázquez Bourgon (Hospital Universitario Marqués de Valdecilla. Santander); Júlia Vendrell Serres (Hospital Universitari Vall d'Hebrón. Barcelona); Iñaki Zorrilla Martínez (Hospital Universitario Araba. Vitoria)
